# Ectogestation and the Good Samaritan Argument

**DOI:** 10.1093/jlb/lsad012

**Published:** 2023-06-07

**Authors:** Christopher Stratman

**Affiliations:** Department of Philosophy and Classics, The University of Texas at San Antonio, San Antonio, TX 78249, USA

**Keywords:** ectogestation, reproductive rights, artificial amnion and placenta technology, pregnancy, abortion, systemic misogyny

## Abstract

Philosophical discussions concerning ectogestation are trending. And given that the Supreme Court of the United States overturned *Roe v. Wade* (1973) and *Casey v. Planned Parenthood* (1992), questions regarding the moral and legal status of abortion in light of the advent of ectogestation will likely continue to be of central importance in the coming years. If ectogestation can intersect with or even determine abortion policy in the future, then a new philosophical analysis of the legal status of abortion is both warranted and urgently needed. I argue that, even if there is no ‘moral’ right to fetal destruction once ectogestation becomes a reality, societies ought not to implement legal prohibitions on a pregnant person’s ability to safely obtain an abortion that results in fetal death because such laws are systemically misogynistic.

## I. THE FUTURE OF REPRODUCTIVE RIGHTS

On Friday, June 24, 2022, the Supreme Court of the United States ruled in *Dobbs v. Jackson* that abortion is not a protected constitutional right.^1^ This incredulous court decision overturned the precedent set by *Roe v. Wade* (1973) and *Casey v. Planned Parenthood* (1992), and in effect nullified a pregnant person’s constitutional right to end their pregnancy.^2^ While it is difficult to forecast what the long-term legal and social consequences will be in the aftermath of *Dobbs*, we have arguably crossed an important threshold into a new era regarding reproductive ethics and rights, insofar as States are now free to implement new abortion bans. And given that this legal sea change will likely culminate in additional threats to core freedoms in the United States, it is acutely important for philosophers, legal scholars, and medical practitioners to examine the role that developments in medical technologies will likely play in our understanding of the moral and legal status of abortion in the future.

Indeed, philosophical discussions concerning ectogestation are trending.^3^ Conceivably, it will eventually (maybe soon) be possible to transfer a fetus from a human womb to a non-human womb where it would be gestated *ex utero* using artificial amnion and placenta technologies (AAPT).^4^ This raises a morally and legally significant question: *if ectogestation is possible, could an unwanted pregnancy be terminated without destroying the fetus in the process and without violating a pregnant person’s right to bodily autonomy?* Indeed, advancements in AAPTs have intrigued philosophers interested in the moral and legal implications involved in this question well before the *Dobbs* ruling.^5^ For instance, some have argued that the possibility of ectogestation shows that there is no ‘moral’ right to the death of the fetus.^6^ Others have argued that there is a right to the death of the fetus.^7^ Indeed, many have already considered the legal implications AAPTs would have for the ethics of abortion.^8^ However, the legal shift that has occurred in the United States as a result of *Dobbs* raises a novel question pertaining to AAPTs: *If there is no ‘moral’ right to the death of the fetus with the advent of ectogestation, does it follow that there must be a corresponding ‘legal’ prohibition on lethal forms of abortion?* My goal in this article is to answer this question by arguing that even if the right to terminate one’s pregnancy does not entail a ‘moral’ right to the death of the fetus, this does not mean that societies ought to implement new legal prohibitions on a pregnant person’s ability to safely secure an abortion that results in fetal death.^9^

The article is organized as follows: I will begin by addressing various important questions that will provide some needed context for the argument to be developed (Section 2). I will then argue that anti-abortion laws are systemically misogynistic and, therefore, immoral (Section 3). This will be followed by a detailed consideration of various objections (Section 4), prior to concluding (Section 5).

## II. IS ECTOGESTATION PLAUSIBLE?

Before moving on, some clarifications regarding ectogestation and the plausibility of this future technology must be addressed, which is the goal of this section.^10^

First, it is crucial to clearly distinguish between several ways of conceptualizing the biological entity that would be gestated in a non-human womb via AAPTs. In the public domain, it is not uncommon to think of the biological entity gestating in the human womb as either a fetus or (for some) a human baby. But some philosophers have claimed that this terminology breaks down in conceptually significant ways regarding the subject of ectogestation. For instance, Romanis claims that the subject of ectogestation is best understood as a ‘gestateling’ rather than a fetus or infant; and this biological entity counts as ‘…a human being in the process of ex utero gestation exercising, whether or not it is capable of doing so, no independent capacity for life’.^11^ And according to Romanis, a gestateling is importantly similar to a pre-viable fetus and, therefore, significantly different from either a ‘newborn’ or a ‘preterm infant’, which is typically construed as a neonate in an incubator.^12^

Other philosophers have objected to Romanis’ distinction. For example, Colgrove argues that Romanis’ distinction between gestatelings and neonates fails for several reasons; the distinction is morally inappropriate insofar as both entities share the same moral status, and since by definition gestatelings count as having been born, then there is no significant difference between the two biological entities.^13^ Clearly there remains various complications regarding this distinction that I cannot canvas in this article, but one of the leading differences that is important to recognize in what follows is whether the biological entity in question should count as viable without the use of AAPTs. For the purpose of this article, however, I will use the common term ‘fetus’ to refer to the entity gestating in a human womb and attempt to remain neutral with regard to the metaphysical details of the entity developing independent of the human womb.^14^

Second, it is common in the literature to distinguish between full and partial ectogestation.^15^ The former refers to the gestation of the biological entity using AAPTs entirely independent of a human womb at every point during its gestation. The latter refers to a future technology, which could presumably allow for the use of AAPTs to extend the viability of the gestating fetus by removing it from a human womb where it is then brought to full term in a non-human womb. Medically speaking, however, the use of AAPTs for partial ectogestation should be construed as sustaining continued gestation only, whereby continued development of vital organs like the lungs and brain would occur.^16^ This is importantly different from our current incubator technologies, which aims only at rescuing a presumed viable fetus that has sufficiently developed in a human womb such that it has a capacity to be born alive.^17^ For the purpose of this article, I will focus entirely on the legal status of abortion given the advent of partial ectogestation.

Third, the term ‘abortion’ is ambiguous: It can refer to an abortion that results in fetal death or it can refer to the termination of one’s pregnancy that does not necessarily entail the death of the fetus.^18^ The advent of ectogestation could conceivably make it possible to terminate one’s pregnancy without necessarily destroying the fetus, though, for some, such an advancement of technology might be hard to imagine. While it might be fairly easy to conceive of a future technology that could extend the age of viability for a fetus slightly (e.g., from 24 weeks to, say, 21 or 20 weeks), it is not at all obvious that such a technology could significantly change the age of viability from, say, 24 weeks to less than 20. Arguably, the age of viability is at least partly determined medically by the development of vital organs such as the lungs. Hence, if a fetus’ lungs have not sufficiently developed, then any future technology must help create a presumed non-viable fetus’ lungs in the process of gestating it in a non-human womb.^19^

While these sorts of worries may be conceptual dead ends for some, for others they are not. I am not making any claim about the likelihood of such technological advancements. Rather, what follows should be understood as a thought experiment, insofar as I am simply stipulating that AAPTs would in fact allow for the possibility of non-lethal abortions given that the following conditions are satisfied: (i) ectogestation would be logistically possible and safe for both the developing fetus and the pregnant person; (ii) ectogestation would be no more invasive than lethal abortions are currently; (iii) ectogestation would be no more socially, economically, or psychologically burdensome to the pregnant person than lethal abortions are currently;^20^ (iv) there would be responsible persons in the moral community who desire and are able to provide the required care for the fetus as it develops into an adult person; and (v) that ectogestation would be adequately funded by societies and equitably distributed across divers socioeconomic and racial populations at least to the same extent that abortion services are currently. Clearly this is a lot to stipulate from the outset. This is why I want to be forthright in claiming that what follows is to be understood largely as a thought experiment.

One could always dispute some or all of these claims on purely practical grounds. For instance, Rodger has argued that the medical procedure required for ectogestation would likely parallel that of various forms of invasive surgeries, as in the case of a cesarean section.^21^ If so, then this would arguably entail an unreasonable risk to the pregnant person, which suggests that (ii) and (iii) are not realistic.^22^ Thus, even if in the future the development of AAPTs reaches a point where (i) is eventually satisfied, it is difficult to think that (ii) and (iii) might ever allow for a plausible alternative to abortions that result in fetal death. Likewise, regarding (iv), one might argue that this claim is unwarranted, insofar as it either flatly ignores the current scarcity of responsible persons in the moral community who would consent to provide adequate foster/adoptive care for such children, or it ignores the current conditions of the foster care system, which are in many instances simply appalling. So, to accept (iv) one would have to assume that the moral community would be conceptually very different than it is currently, such that responsible people exist and can be identified who would in fact take on the social and economic obligations/challenges of raising a child to adulthood.

Furthermore, given both the historical inequities across divers socioeconomic and racial populations associated with abortion services (at least in the United States) and the expected future inequities post *Dobbs*, (v) is arguably the most palpably problematic of these claims.^23^ Indeed, many have argued that abortion services ought to remain legal notwithstanding the introduction of AAPTs that could allegedly avoid any violation of a pregnant person’s right to bodily autonomy.^24^ But it is worth mentioning that philosophers who deny (v) typically do so on the grounds that there is a ‘moral’ right to the death of the fetus. In what follows, I will take a different approach, which can avoid the pressures to deny (v)—that is, my view says that even if there is no ‘moral’ right to fetal destruction, given the advent of ectogestation, this does not mean that societies ought to implement any new legal prohibitions on a pregnant person’s ability to obtain an abortion that results in fetal death.

Now, while I recognize why one might be tempted to reject (i)–(v) above, I want to put the anti-abortion position on the firmest ground possible. So, I will not attempt to defend or reject these claims.^25^ Rather, I will simply stipulate that (i)–(v) are plausible for the sake of this article. Thus, once again, I want to be plainspoken in acknowledging that, if you prefer, what follows can be construed as nothing more than a kind of mere thought experiment. But this still assumes that the above stipulations are true in order to then show that this fact alone does *not* entail (logically, conceptually, pragmatically, etc.) that societies ought to implement new legal restrictions on a pregnant person’s ability to safely obtain an abortion that results in the death of the fetus.^26^

## III. THE GOOD SAMARITAN ARGUMENT

The goal of this section is to show that the anti-abortion position should be rejected because it is inherently misogynistic and immoral. If this is right, then we should resist the view that if ectogestation is possible, then societies ought to implement new legal prohibitions on a pregnant person’s ability to safely obtain an abortion that results in fetal death. The argument I will defend is not new but it is extremely powerful.

To my mind, Thomson’s (1971) insightful distinction between *Minimally Decent Samaritan Laws* and *Good Samaritan Laws* generates an intuitively compelling reason to conclude that even if it is true that ectogestation would allow for a fetus to be safely removed from a human womb and transferred to a non-human womb without destroying it, this would not justify (legally or morally) the implementation of new prohibitions on a pregnant person’s ability to obtain an abortion that results in fetal death.^27^

Recall what Thomson claims regarding Good Samaritan laws: ‘I should think, myself, that Minimally Decent Samaritan laws would be one thing, Good Samaritan laws quite another, and in fact highly improper’.^28^ The reason for this opinion is revealing and instructive. So, I want to quote what Thomson said in detail:

Indeed, with one rather striking class of exceptions, no one in any country in the world is legally required to do anywhere near as much as this for anyone else. The class of exceptions is obvious. My main concern here is not the state of the law in respect to abortion, but it is worth drawing attention to the fact that in no state in this country is any man compelled by law to be even a Minimally Decent Samaritan to any person…By contrast, in most states in this country women are compelled by law to be not merely Minimally Decent Samaritans, but Good Samaritans to unborn persons inside them. This doesn’t by itself settle anything one way or the other, because it may well be argued that there should be laws in this country—as there are in many European countries—compelling at least Minimally Decent Samaritanism. But it does show that there is a gross injustice in the existing state of the law. And it shows also that the groups currently working against liberalization of abortion laws, in fact working toward having it declared unconstitutional for a state to permit abortion, had better start working for the adoption of Good Samaritan laws generally, or earn the charge that they are acting in bad faith.^29^

Building on these remarks, one could reasonably maintain that any new attempts to restrict a woman’s ability to safely and lawfully obtain a lethal abortion, though non-lethal means of terminating unwanted pregnancies may be an available alternative, would in effect be a law compelling people with uteruses to be Good Samaritans.^30^ And if such laws would be grossly unjust, as Thomson suggests in the above passage, then surely this would provide the basis for an argument that purports to show that such legal prohibitions are systemically misogynistic. I will call this the ‘Good Samaritan Argument’:

### III.A. The Good Samaritan Argument

(1) If a law is harmful, then societies ought not implement it.
(2) If societies ought not implement a harmful law, then it would be morally wrong if societies do implement such a law.
(3) If a law is harmful, then it would be morally wrong if societies implement such a law.^31^
(4) Good Samaritan laws are systemically misogynistic.
(5) Systemically misogynistic laws are harmful.
(6) Thus, Good Samaritan laws are harmful.^32^
(7) Therefore, it would be morally wrong if societies implement Good Samaritan laws because they are systemically misogynistic and harmful.^33^

One powerful reason for why it seems so intuitively compelling to claim that anti-abortion groups working to implement prohibitions on lethal abortions are acting in bad faith, as Thomson suggests, is because such laws are inherently unjust and immoral regarding their treatment of people with uteruses. Even still, this is not because they (of necessity) foster immoral or unlawful violations of a right to bodily autonomy as it is often thought.^34^ Given that AAPTs could make possible ectogestation, then, arguably, the fetus could continue to be gestated independently of the pregnant person’s womb without violating their right to bodily autonomy.^35^ But, if it turns out that anti-abortion laws are not unjust and immoral in virtue of violating a pregnant person’s right to bodily autonomy, then in virtue of what are such laws unjust and immoral?

Here is one likely answer: Anti-abortion laws are systemically misogynistic, insofar as the primary aim of such laws is to police, regulate, and punish ‘bad’ women who either implicitly or explicitly betray a kind of socially constructed trope, which says that a part of what it means to be a woman is essentially tied to one’s capacity to be a baby-maker and governess. For instance, according to one study, women who identify as Pro-Life are more likely to believe that ‘*good women* make motherhood their top priority’, whereas women who choose not to pursue motherhood above all else ‘were constantly cited as examples of women with the sort of inadequate value systems that lead them to value the material aspects of life above their role, as mothers…’.^36^ As Cannold further observes regarding this attitude toward so-called ‘good’ mothers commonly held by Pro-Life women:

A good mother values her role, or her potential role, as a mother beyond all other aspects of life, placing her children, and potential children, above her own interests, ambitions and goals as an autonomous human being. For a good mother, in other words, there is no such thing as an *unwanted* pregnancy, only an *unexpected* one. A good mother’s response to unexpected pregnancy is a willingness to make room in her life for the new arrival (p. 62; emphasis in the original).^37^

Now, given that there is this widespread assumption in society that a ‘good’ woman is essentially a caregiver, it is not at all surprising that anti-abortion laws are inherently misogynistic.^38^ If this is correct, then it is reasonable to think that we ought not implement new prohibitions on lethal abortions because to do so would be to further support, encourage, and give legitimacy to systemic misogyny.^39^ Indeed, the astute view is that governments and citizens ought to resist any attempt to implement laws rooted in systemic misogyny. Hence, we have *prima facie*, defeasible support for the claim that, given the advent of ectogestation, societies should not implement new legal prohibitions on a pregnant person’s ability to safely obtain an abortion that results in fetal death.

Now, proponents of the anti-abortion position will likely object by claiming that this is an obvious *ad hominem* against Pro-Lifers. Indeed, one might argue in the following way: There are many Pro-Lifers who believe that it is perfectly acceptable for women not to be baby producers or caregivers. Are we really supposed to believe that if you are Pro-Life, then you cannot fail to hate women? Or if you are Pro-Choice, then you are thereby immune to misogynistic beliefs? Such a position is utterly unpalpable and absurd.

Notice that this objection is effective only if the notion of misogyny is construed in terms of a belief held by individuals or particular agents in a society. But as Manne (2018) has argued regarding this naïve understanding of misogyny, this view is mistaken. Misogyny is not a mental attitude that an individual (often a man) bears, which disposes them to ‘feel hatred, hostility, or other similar emotions toward any and every woman, or at least women generally, *simply because they are women*’.^40^ According to Manne, there is a more accurate way of thinking about the nature of misogyny. Here is Manne’s ‘ameliorative’ analysis of the term:

According to the ameliorative feminist conception of misogyny that I motivate… misogyny is primarily a property of social systems or environments as a whole, in which women will tend to face hostility of various kinds *because they are women in a man’s world* (i.e., a patriarchy), who are held to be failing to live up to patriarchal standards (i.e., tenets of patriarchal ideology that have some purchase in this environment).^41^

But why should we accept Manne’s analysis of misogyny rather than the naïve definition? Since Manne offers a persuasive reason to forsake the naïve conception of misogyny, I will quote the passage at length. Manne says:

I believe that the naïve conception of misogyny is too narrow in some respects and not focused enough in others. Although I think it is right to keep the emphasis on attitudes in the family of hostility, I argue that the targets of this hostility should be allowed to encompass *particular* women and particular *kinds* of women. Otherwise, misogyny will be effectively defined so as to be rare in patriarchal settings—which I take to be its native habitat—given certain truisms about the moral psychology of hostility and hatred. The naïve conception also fails to home in on the subclass of these reactions that I think deserve to be our focus here: those that are an out-growth of *patriarchal ideology*. For misogyny, though often personal in tone, is most productively understood as a *political* phenomenon. Specifically, I argue that misogyny ought to be understood as the system that operates within a patriarchal social order to police and enforce women’s subordination and to uphold male dominance.^42^

Indeed, this conception of systemic misogyny has a clear advantage of being able to explain the otherwise inexplicable fact that there exist individuals (men and women) in society who profess to hold egalitarian views but nevertheless implicitly endorse or engage in misogynistic behavior. I take these reasons to be a powerful motivation for why one might endorse Manne’s proposal.^43^

Now, if what Manne says about systemic misogyny is plausible, then the mere possibility of ectogestation would not be sufficient grounds to restrict a pregnant person’s lawful ability to safely obtain a lethal abortion. And this is precisely because such laws are essentially rooted in a social-political supervisory function whose primary purpose is to punish pregnant people who fail to conform to the social–political norms and expectations of that patriarchal system. Indeed, such laws are in effect a form of gaslighting, which aims at preserving the authoritative narrative told by the patriarchal collective. Thus, we have good grounds to refrain from and actively resist implementation of new legal prohibitions on lethal abortions, insofar as laws of this sort are birthed from an unjust and immoral systemically misogynistic womb.

Arguably, however, the most contentious premise is (6)—the claim that Good Samaritan laws (e.g., abortion bans) are harmful. While some may find this premise intuitive and, perhaps, obvious, proponents of abortion bans will likely object by arguing that it is unsupported. So, it is imperative to demonstrate why this claim is true.

First, systemic misogyny permeates every aspect of modern society and our lived experience whether we realize it or not (e.g., misogyny is deeply intertwined in our language, public discourse, television, the movie industry, advertisements, our online experiences, and social media). As Tirrell observes: ‘Misogynist discourse, in speech and images, inflicts tremendous harm on women every day, reinforcing lower social status, and rationalizing discrimination and mistreatment—including unequal pay, sexual abuse and violence, and more’.^44^ And according to a (2021) survey, 82 per cent of young women ages 11 to 21 routinely experience harmful content online; 50 per cent of this harmful content were comments in sexual or sexist nature, while 28 per cent was described as harassment and 21 per cent was described as bullying.^45^ Indeed, the anonymity and ubiquitous nature of the internet and social media raises deep and wide-ranging challenges regarding the harms of misogyny in modern society.

While it may seem easy to show the profound harms of systemic misogyny that occur in online spaces, like picking low hanging fruit, the harms of misogyny can and do happen everywhere. Consider, for example, the following experiences of harmful attacks reported by various women and collected by The Working Group on Misogyny and Criminal Justice’s Independent Report (2022):

Since I have become disabled this [harassment] happens so much more. I had a man on a train telling me ‘ooh love did someone fuck you too hard and now you’re broken?’ I was so scared.

Thirteen year old school boys followed a thirteen year old school girl asking what sexual position she liked, how many times she had had sex… When she refused to answer they called her a slut and told her she didn’t deserve to breathe the same air as men. This was on the school playground… I reported it only to be told ‘boys will be boys… it’s just banter… they will grow out of it’.

Being threatened by a man in a pub for not laughing at what he thought was a funny remark.

Myself and a female partner were out running and a group of young people were walking towards us… one of the young men started shouting… specifically lesbophobic misogyny—so shouting ‘fucking lezzies’.^46^

The unfortunate fact is that these sorts of reported cases of misogyny are not uncommon.^47^ And the harms ensued by those who undergo such experiences are probably far more extensive than most are willing to admit or can imagine. As Tirrell (2019) has argued:

The daily onslaught of misogynist speech and images endangers women and causes significant social, psychological, and economic harms to both our sense of well-being and our actual safety and development. Ubiquitous in patriarchal societies, misogyny is inherent in the structural norms that shape gender identities, relationships, economics, politics—it is woven into the very fabric of society.^48^

Indeed, the omnipresent nature of the patriarchal structures at the core of so many cultural expectations and social norms can make it difficult for some to truly recognize such experiences as real harms because of the sheer pervasiveness of systemic misogyny. Of course, the proponent of legal prohibitions on lethal abortion could conceivably accept that systemic misogyny is an evil to be avoided but still maintain that unless it is shown that misogynistic *laws* are harmful, the Good Samaritan Argument is unsupported. So, let us consider some examples where it should be easy to recognize the harms that result from misogynistic laws.

Much attention has recently been given to Iran’s compulsory hijab laws or forced veiling laws as a result of the arrest and death of Mahsa Amini on September 13, 2022. Amini’s death sparked the organization of numerous protests that have been ongoing for months.^49^ But what exactly are these brave individuals protesting? On the one hand, they are protesting Amini’s arrest and murder by the hands of the so-called ‘morality’ police in Tehran, for what is obviously a misogynistic law.^50^ On the other hand, these brave souls are not just protesting the treatment of Amini (her arrest and subsequent murder), they are protesting the profusion of misogyny that saturates so many parts of Iranian society and is evidenced by laws that enforce the veiling of girls and women.^51^ Indeed, over 40 million girls and women live under daily surveillance by the government.^52^ Waiting and watching for any sign of an offense or violation of the patriarchal expectations and social norms, the government, entirely made up of men, control every aspect of a girl or woman’s life in Iran. This is not merely a kind of hatred of women or belief that women are inferior to men expressed by the beliefs of certain people in power. It is systemic misogyny expressed in the legal system and institutions of Iran. Thus, the harms endured by millions of people in Iran are the direct result of systemic misogyny understood as Manne defines it: ‘the system that operates within a patriarchal social order to police and enforce women’s subordination and to uphold male dominance’.^53^ Likewise, the misogyny at the root of Iran’s compulsory hijab laws is clearly harmful to girls and women, such that any attempt to maintain otherwise should not be taken seriously.

But what about laws that ban abortion? What reason is there to think that these sorts of legal prohibitions are harmful? Until recently, folks in the United States might understandably fail to recognize the harms that result from such laws, insofar as one would have to either consider laws that predate *Roe* or laws in jurisdictions outside of the United States and most European countries. But in a post-*Dobbs* reality, it is myopic to deny the harms of abortion bans. For instance, people in need of cancer care already face numerous therapeutical limitations if they become pregnant.^54^ And given that people of color tend to have a much shorter rate of survival and a much higher rate of mortality from cancer than other ethnic groups, if they become pregnant, they will also be disproportionately affected.^55^ So, one clear consequence of implementing bans on abortion is that it generates medical dilemmas that literally did not exist prior to *Dobbs*. Hence, States where legal prohibitions on abortion now exist needlessly create life-threatening harms for cancer patients and people of color. Here is how one medical professional describes the consequences of bans on abortion for cancer patients:

Put simply, states that have enacted or have proposals to enact laws classifying fertilized eggs, zygotes, embryos, and fetuses as having full legal protections from conception create a likely barrier for a subset of pregnant women to receive immediate, effective cancer care. This governmental policy creates, for the first time in the modern era of oncology, insertion of government into the cancer care decision-making for pregnant patients (Knudsen, 2022).^56^

It seems difficult to deny that the consequences of *Dobbs* are both heartbreaking and lethal. But for the purpose of seeing why abortion bans are harmful, it is revealing to think about how the harms of abortion bans affect more than just people with uteruses.^57^ The realities of a post-*Dobbs* United States ensure that physicians will arguably not be sufficiently well-informed regarding what is legally permitted in the State where they practice.^58^ And given that *Dobbs* directly impacts the medical decisions that physicians routinely face, and granting that at least some of these physicians are men, it seems reasonable to think that they might also be at least indirectly affected by the potential harms of new prohibitions on abortion. While such harms may not directly threaten their life, they would undoubtedly have to needlessly reevaluate their approach to medicine, which would very likely impact their general wellbeing.^59^ Moreover, insofar as people who need cancer treatment might become pregnant and might already be parents, their children might also be indirectly harmed as a result of banning abortions. While it may be tempting to focus on the harms that would directly affect only pregnant people, it would be a mistake to conclude that only they would be harmed. Thus, it is implausible to think that abortion bans do not cause serious harms.

Furthermore, since the summer of 2022 when *Dobbs* became United States law, numerous articles have been published discussing the harms for pregnant people that arise as a direct result of this decision.^60^ For instance, according to one such story, a Texas woman was legally forced against her will to carry her dead fetus for several weeks because, though the fetus was no longer living, all abortion services were banned.^61^ And according to another story, a 10-year-old girl, who had become pregnant as a result of rape, was forced against her will to travel from Ohio to Indiana to receive an abortion because all abortion services had been banned in the State where she lived.^62^ These sorts of cases are unfortunately now a medical reality that millions of people have no choice but to endure in a post-*Dobbs* United States.

Now, I fully admit that this is only a gloss on the ways in which abortion bans are harmful. Nevertheless, I maintain that it is implausible to think that cases such as these would not count as serious harms resulting (directly or indirectly) from laws banning abortion. So, if the proponent of anti-abortion laws is to show that such laws are *not* harmful, they would have to demonstrate that one or both of the following claims are true: (i) the sorts of harms that occur in the examples described above are not the result of abortion bans, or (ii) the sorts of harms that occur in the above examples would still occur even if the relevant bans on abortion were not in place. But, neither of these claims are even remotely plausible. Hence, we have excellent grounds to conclude that, even once ectogestation becomes a reality, societies should not implement legal prohibitions on a pregnant person’s ability to safely obtain an abortion that results in fetal death because such bans are misogynistic and immoral.^63^

## IV. POTENTIAL OBJECTIONS CONSIDERED AND DISMISSED

I have argued that abortion bans are systemically misogynistic and harmful. Thus, societies should not implement legal prohibitions on a pregnant person’s ability to safely obtain an abortion that results in fetal death, and this extends to future situations where AAPTs have made ectogestation a reality. It is now time to consider several important objections to the argument I have defended, which is the goal of this section.

*First Objection*: Some might agree that anti-abortion laws are immoral and should not be implemented by societies given the advent of ectogestation, but argue that this is because there is a right to the death of the fetus. Indeed, someone might argue that showing that there is a more fundamental right to secure the death of the fetus is a necessary step in showing that anti-abortion laws are systemically misogynistic. Hence, my interlocutor might claim that what I have argued presupposes that there is a moral right to the death of the fetus.

*Response*: Is there a right to the death of the fetus? Three arguments have been offered in support of the view that such a right exists and is grounded in a more basic right: (i) a right to avoid being or remaining a parent; (ii) a right to genetic privacy; (iii) a right to property.^64^ Numerous articles have been authored in recent years attempting to show why each of these arguments fail to demonstrate that there is a right to secure fetal death.^65^ So, I will not endeavor to further contribute to this debate. However, for the purpose of responding to this objection, I want to make clear that if any of these arguments are successful, then the right to the death of the fetus would probably be a collective right that both of the biological parents possess jointly.^66^ While I am not convinced that there is a collective right to the death of the fetus, it is not obviously false that such a right might exist. So, let me explain why I think there is *prima facie* reason to be skeptical of an alleged collective right to the death of the fetus.

As things currently stand, the presumption is that the right to terminate one’s unwanted pregnancy is not construed as a collective right shared by both of the biological parents but an individual right that the pregnant person alone possesses. So, if the right to the death of the fetus is to be understood as a collective right, then the burden of argument is on those who assert that it is not an essentially distributive right possessed by the pregnant person alone. Moreover, it is widely accepted that rights are possessed individually, not collectively. For instance, regarding the evident accord on this matter, Wellman (1995) writes: ‘If there is any consensus in these very difficult conceptions of rights, it is that the concept of a right is essentially distributive, that is, rights are ascribed to and possessed by each individual or entity in a group separately rather than collectively’.^67^ Thus, if the best versions of the arguments mentioned above require interpreting the right to the death of the fetus in terms of a collective right, then we would have *prima facie* evidence for why these arguments each fail to demonstrate that there is a right to the death of the fetus, since it is not at all clear that rights are not essentially distributive and individually possessed. While this evidence is admittedly defeasible, I take it that the objection can be avoided, insofar as the Good Samaritan argument does not hinge on whether there is a right to the death of the fetus or not. Indeed, this response is well supported by Thomson’s observation that one would not be guaranteed a right to destroy the famous violinist even if he somehow is able to survive being detached.^68^

*Second Objection*: Many have argued that if the advent of ectogestation means that there is no right to the death of the fetus, then this would be sufficient to conclude that societies should implement legal prohibitions on a pregnant person’s ability to safely obtain an abortion that results in fetal death.

*Response*: I think this objection can be avoided because even if ectogestation would ground a reason to think that there would be fewer lethal abortions, this would probably not count as the very same reason that would be needed to conclude that societies ought to enforce laws that restrict lethal abortions. But in order to make this point, I must first explain why some accept this view. Consider the following argument, which I call the ‘Anti-Abortion Argument’:

### IV.A. The Anti-Abortion Argument

(1) Given the possibility of ectogestation via AAPTs, a pregnancy can be terminated by safely transferring a fetus from a human womb to a non-human womb without killing the fetus or violating a pregnant person’s moral right to bodily autonomy.
(2) If a fetus can be safely removed from a human womb and gestated in a non-human womb without killing it or violating a pregnant person’s right to bodily autonomy, then there is no moral right to the death of the fetus.
(3) If there is no moral right to the death of the fetus, then new legal prohibitions on one’s ability to safely and lawfully secure the death of the fetus ought to be implemented.
(4) Therefore, new legal prohibitions on lethal abortions ought to be implemented.

To my mind, this is a bad argument, though it is not obviously bad. In order to evaluate why this is a bad argument, let us first consider why someone might accept (1) and (2).

Proponents of the Anti-Abortion argument typically ask us to imagine a future situation where advancements in AAPTs have progressed to a point where ectogestation is a safe alternative to lethal abortions.^69^ As Kaczor claims: ‘The “win-win” scenario of artificial wombs rather than ending the life of the prenatal human being has been endorsed by a number of authors on all sides of the debate’.^70^ Indeed, it is not conceptually incoherent to think that someone might embrace ectogestation for various reasons. But the core of this argument seems to be the idea that AAPTs have the potential to profoundly alter peoples’ views about the moral and legal status of abortion. For instance, Mathison and Davis (footnote 2) write: ‘we believe that the possibility of ectogenesis brings about a unique way of framing the question of the right to the death of the foetus that does not require settling the question of the right to bodily autonomy’.^71^ Similarly, Blackshaw & Rodger claim: ‘Any arguments for abortion that rely on the right to bodily autonomy or self-defense will be impacted, shifting the debate to the question of whether there is a right to the death of the fetus’.^72^ Of course, someone might object by arguing that, even if AAPTs can provide a safe way to terminate unwanted pregnancies without killing the fetus, to limit the medical procedures available to a pregnant person who desires to end their unwanted pregnancy would still be a violation of bodily autonomy.

But this objection misrepresents what it means to have a right to bodily autonomy—that is, it conflates the right to terminate an unwanted pregnancy with a right to secure the death of the fetus. As Warren tells us regarding the fate of an unwanted fetus: ‘While she might prefer that it die, rather than being raised by others, it is not clear that such a preference would constitute a right on her part’.^73^ Said differently, it is one thing for the right to bodily autonomy to be understood as a guarantee that, if the pregnant person chooses, the unwanted pregnancy can be terminated; it is quite another thing to interpret this right as a right to the death of the fetus. Thus, to show that the right to bodily autonomy entails a right to the death of the fetus, one must first deliver independent grounds for the claim that there exists a right to the death of the fetus.^74^ To assume without argument that the notion of abortion conceptually entails or simply means that a pregnant person has a right to the death of the fetus would beg the question. It is this further claim regarding whether there is a right to the death of the fetus, once AAPTs allow for the possibility of ectogestation, that is disputed on both sides of the debate.

But the problem with the anti-abortion argument offered above is not whether there is anything like a ‘moral’ right to the death of the fetus—we can simply stipulate for the moment that there is no such right. The real problem with the argument is the third premise. Even if there is no ‘moral’ right to the death of the fetus, it does not follow that societies ought to implement new legal prohibitions on a pregnant person’s ability to safely and lawfully secure the death of an unwanted fetus? Of course, some have suggested that without a ‘moral’ right to the death of the fetus, societies should implement new prohibitions on one’s ability to obtain a lethal abortion.^75^ But it is not implausible to think that a society might *not* implement new legal prohibitions on safe and lawful lethal abortions if there are independent reasons for why a society would decide against implementing such laws. For instance, if the foster-care or adoption systems in the relevant society were corrupt and below a minimal level of acceptability for a life to go well, this might ground a cogent reason not to implement new prohibitions against lethal abortions. If this is plausible, then (3) would either be false or unsupported. Thus, one would need a further argument to derive the conclusion from the premises—that is, what is needed is a modification, such that (3) is replaced with the following:

(3a) If the lack of a moral right to the death of the fetus is sufficient to restrict a pregnant person’s ability to safely obtain a lethal abortion, then new legal prohibitions on lethal abortions ought to be implemented.

(3b) The lack of a moral right to the death of the fetus is sufficient to restrict a pregnant person’s ability to safely obtain a lethal abortion.

But if either of these premises turn out to be false, this will be sufficient to reject the argument. The problem here is that, while it may be tempting to think that the advent of ectogestation would give us a reason to conclude that there would probably be fewer abortions that result in fetal death, this alone would not show that, legally speaking, there ought to be new laws prohibiting lethal forms of abortion. One would need an additional argument to make the move from (3b) to (4).

I will grant that advancements in AAPTs in fact *will* give us a good reason to think that there will be fewer abortions that result in fetal death.^76^ But even if this assumption is true, it is not clear whether this reason would itself be *the very same reason* to think that we ought to implement new legal restrictions on one’s ability to secure the death of the fetus. In order to show that the lack of a moral right to the death of the fetus is sufficient to restrict a pregnant person’s ability to safely obtain a lethal abortion, it must be shown that the reason R (i.e., why there would be fewer lethal abortions) is the very same reason R (i.e., why we ought to legally prohibit lethal abortions), rather than a different reason R*. But this is precisely what I have claimed is false. And I have offered a principled reason for why lacking a ‘moral’ right to the death of the fetus is not sufficient to restrict a pregnant person’s ability to safely obtain a lethal abortion once the arrival of ectogestation occurs. Namely, Good Samaritan Laws are systemically misogynistic and harmful.

Still, someone might respond by arguing that even if it is true that lacking a ‘moral’ right to the death of the fetus is not sufficient to conclude that we ought to implement new legal restrictions on a pregnant person’s ability to safely and lawfully obtain an abortion that results in fetal death, once AAPTs make ectogestation a safe and widely available alternative, surely it must be a necessary condition. And if it is a necessary condition, then, arguably, one could still maintain that because the state has an interest in protecting the life of the fetus, the default position should be to implement legal restrictions on a pregnant person’s ability to secure the death of the fetus.^77^

But taking this position would arguably concede far more than it could achieve. It assumes that the default position must be to protect the life of the fetus at the cost of accepting the inexorable logic that the argument would then be unsupported. Perhaps the objection will succeed in showing that the opposing positions result in an impasse. But even this is too reconciliatory. While it may be true that the State has some interest in protecting the life of the fetus, the State also has an interest in uprooting, rescinding, and actively working to repeal those aspects of society that are systemically misogynistic. And it is entirely plausible that the State has a superior obligation to those people who are the targets of systemic misogyny than fetuses, even granting that the State has some interest in the lives of fetuses. To put it a bit more crudely, surely the State ought to have an interest in ceasing the maltreatment of roughly half of its actual citizens, even at the cost of its interest in preserving the life of its potential citizens. Hence, this objection can be avoided.

*Third Objection*: Perhaps (3b) in the Anti-Abortion Argument could be defended by appealing to the notion of viability as a kind of legal threshold for determining abortion laws. If so, then, presumably, one could show that societies have a good reason to implement prohibitions of lethal abortions independent of whether the advent of ectogestation gives us a reason to think that there would be fewer lethal abortions. The idea is this: If AAPTs could successfully extend the age of viability of the fetus (currently at approximately 24 weeks), then this could arguably provide an independent *reason* for why societies should implement new legal restrictions on a pregnant person’s ability to safely obtain an abortion that results in fetal death.

*Response*: This objection is problematic in several ways. First, the objection assumes that the notion of viability is coherent and consistently applied across various legal jurisdictions. But as we shall see, this assumption is probably false. Second, the objection assumes that the notion of viability is a legally coherent standard widely adopted by courts to determine whether fetal destruction should be legally permitted or not. But, at least in the United States, *Dobbs* has radically altered the legal landscape such that many States no longer use the age of viability as a legal threshold. Third, even if the concept of viability is legally coherent, it remains an open question whether or not the courts would in fact use this concept as a legal threshold given the range of ways the concept has been applied across diverse legal jurisdictions. And if any of these responses are plausible, this will suffice to show that the objection can be avoided. Let us consider each in turn.

To my mind, Romanis (2020) offers a compelling case for why the notion of viability is at best vague and not well understood, and at worst incoherent and inconsistently applied across multiple legal jurisdictions. The basic idea behind Romanis’ argument is this: In England and Wales, the notion of fetal viability is imprecise by design so as to allow physicians a suitably wide range of possible interpretations depending on the medical details of the relevant situation.^78^ The primary point of contention in the current law in England and Wales hinges on what it means for a fetus to have a ‘capacity to be born alive’. And according to Romanis’ analysis, this is not a virtuous way to ‘resolve the issue of whether the capacity to breathe only for a short time after birth would be sufficient, or whether the capacity would have to be more substantial (such as longer-term use of the lungs)’.^79^ On the other hand, in the United States the legal understanding of viability has shifted in numerous ways and continues to vary from State to State. For instance, in *Casey*, the United States Supreme Court claimed that fetal viability ought to track any relevant technological advancements that could make a difference for prenatal care.^80^ Hence, quite literally, the legal meaning of the concept ‘fetal viability’ is partly determined by an unstable and forever changing technological landscape.

The inconsistencies that Romanis identifies is made all the more difficult by the fact that the court has in effect punted the responsibility of giving a precise and legally meaningful point of viability to each of the States to decide. As Romanis observes: ‘The Supreme Court’s refusal to quantify viability means that States have been left to define viability as they see fit, which has resulted in a multitude of approaches’.^81^ Romanis suggests that there are three general types of regulations that are taken by the States:

Regulations at State level largely fit into three categories: those that define viability as a matter of medical judgement; those that define viability by referencing the capacity or features of the fetus; and those that define viability as a fixed point in gestation.^82^

*Prima facie*, these categories are inconsistent. And they are arguably motivated by political goals at the State level rather than medical realities concerning prenatal care. Indeed, the inconsistency gets worse once we add England and Wales into the mix since this fourth category would bring vagueness by design into the fold. Moreover, the concept is not only legally incoherent and inconsistently applied; in various jurisdictions, fetal viability has been abandoned as a meaningful legal standard for determining abortion laws.

Interestingly, Romanis observes that in 2019 many States began to favor heartbeat laws over the viability framework entirely.^83^ Generally speaking, these laws criminalize any abortions performed after a fetal heartbeat is detected, which typically occurs approximately 6–8 weeks after conception. Romanis makes clear that, at the time, the Supreme Court had blocked each of these laws from going into effect.^84^ But, since *Dobbs* overturned *Roe v. Wade* in the summer of 2022, many of these laws automatically went into effect as a direct result.^85^ So, Romanis was correct in claiming that the mere fact that these States passed these so-called ‘trigger laws’ makes it clear that there is ‘a political determination among some legislatures to challenge *Roe v Wade*’.^86^ And the fact that these heartbeat laws are now the law of the land in many States arguably repudiates the claim that viability can serve as a plausible legal threshold for determining abortion laws.^87^

Finally, if the legal meaning of viability is inconsistent across numerous legal jurisdictions, and if some States have abandoned viability as a legal threshold for determining abortion law, then why would a court use the concept of viability given the advent of ectogestation? At best, this seems to be an open question. Let me explain.

Consider the Abortion Act (1967) introduced by the Parliament in the United Kingdom. First, this piece of legislation codified existing case law recognizing that abortion is a crime, but establishes the legal grounds for when such a crime is *not* committed. In this regard, then, the Abortion Act is a statute that attempts to make clear the legal justification for lethal abortions and has jurisdiction in England, Wales, and Scotland, but not in Northern Ireland. In addition, it extended the legal grounds for the lawful termination of unwanted pregnancies. According to the Abortion Act, there are four broad grounds for a lawful abortion that results in the death of the fetus that are open to differing interpretations. Generally speaking, these are captured by the idea that a lethal abortion is legally acceptable only when the furtherance of a pregnancy is deemed by two registered medical practitioners who ‘in good faith’ concur that it is warranted.^88^ But such grounds must conform to the following:

(a) that the pregnancy has not exceeded its 24th week and that the continuance of the pregnancy would involve risk, greater than if the pregnancy were terminated, of injury to the physical or mental health of the pregnant woman or any existing children of her family; or

(b) that the termination is necessary to prevent grave permanent injury to the physical or mental health of the pregnant woman; or

(c) that the continuance of the pregnancy would involve risk to the life of the pregnant woman, greater than if the pregnancy were terminated; or

(d) that there is a substantial risk that if the child were born it would suffer from such physical or mental abnormalities as to be seriously handicapped.^89^

To my mind, the motivations underlying the Abortion Act help to explain why someone might accept (3b)—the claim that lacking a moral right to the death of the fetus is sufficient to restrict a pregnant person’s ability to safely obtain a lethal abortion. As Rodger (2020) suggests:

Unless it is medically indicated, women do not and are not legally permitted to request to deliver their foetus prematurely at the point of viability (24–28 weeks) with the intent that the child receive neonatal intensive care under the responsibility of adoptive parents. If ectogestation is to function as an alternative to abortion, this is what women would be expected to do.^90^

But in order to unpack this point, let us consider a series of cases where a pregnant woman, in agreement with two registered medical practitioners and two adoptive parents, elects to utilize AAPTs in the form of ectogestation.^91^

*Rose*: Suppose that Rose is experiencing an unwanted pregnancy and has decided to give the child up for adoption once delivered. Let us say that the Smiths are the adoptive parents who have agreed to take responsibility of the child once it is born. Now, imagine that the following extremely rare situation occurs. In the 24th week of pregnancy (when the fetus is considered viable), the health and wellbeing of Rose becomes a medically significant issue, such that the continuance of the pregnancy would involve serious risk to the life of Rose, greater than if Rose’s pregnancy were terminated. The question I want to raise is this: Would it be morally or legally acceptable for the fetus to be surgically removed from the human womb and gestated to full term using AAPTs, if all parties (i.e., Rose, the Smiths, and two registered medical practitioners) agree that this is the best course of action for the health and wellbeing of Rose? This case is a paradigm example of the grounds the Abortion Act says would justify terminating one’s pregnancy. Rose cannot legally opt out of the pregnancy merely because she might not want to be pregnant. The fact that Rose’s life is at risk in this case is the *only* legal grounds for terminating the pregnancy. But, given that the only salient difference is that the use of AAPTs at the 24th week means that the fetus need not die in the course of terminating Rose’s pregnancy, intuitively it is entirely reasonable to conclude that utilizing AAPTs to preserve the life of the fetus would be morally and legally permissible.^92^

*Roslyn*: Now, hold everything fixed save a single feature: Suppose that it is Roslyn’s mental health and wellbeing that is at risk. Perhaps Roslyn is experiencing severe depression and has expressed thoughts of completing suicide, such that the risk is greater than if the pregnancy were terminated. Just as in the previous case, let us suppose that all morally and legally relevant parties agree that the best course of action is to surgically remove the fetus from the human womb where it will be gestated using AAPTs in the form of ectogestation.^93^ Would it be morally or legally permissible under the Abortion Act for this to occur? Presumably, yes. The law explicitly says that the mental health of the pregnant woman may be a legitimate factor in the decision to terminate one’s pregnancy. So, if the fetus can be safely removed from the human womb, gestated using AAPTs, and all relevant parties agree that this is the best course of action, then it is hard to see what might justify prohibiting Roslyn from undergoing this procedure.^94^

*Rosie*: Again, let us hold everything else fixed. But let us stipulate that the continuance of the pregnancy does *not* involve a risk to Rosie, greater than if the pregnancy were to be terminated. In such a case, would it be morally or legally permissible for Rosie to elect to undergo a surgical procedure at 24 weeks, once the fetus is viable, whereby the fetus would be removed from the human womb, gestated in a non-human womb using AAPTs, with the intention of the Smiths taking over legal guardianship including all parental responsibilities of the fetus? It is not immediately obvious whether this course of action would be immoral or not. Presumably, one could appeal to Rosie’s fundamental ‘moral’ right to bodily autonomy in arguing for why this sort of case should count as morally permissible. Likewise, it is not implausible that this course of action would fail to be legally permissible on grounds that neither Rosies’ mental or physical health and wellbeing are in jeopardy. But it *is* likely that the fetus’s health and wellbeing would be at risk, greater than if the pregnancy was not terminated, given currently existing incubator technology.

But what about a hypothetical future scenario where AAPTs have become so advanced that they are commonly used at the 24th week, assuming that the fetus is viable, where the fetus is gestated to full term? In such a scenario, it is plausible to think that if Rosie were to elect to undergo a procedure whereby, at 24 weeks, the fetus is surgically removed from the human womb and gestated in a non-human womb using AAPTs, this would be morally permissible.^95^ And if the Smiths agree to take over the legal guardianship of the fetus at 24 weeks, this too seems morally acceptable. If this is plausible, then it should likewise be plausible if AAPTs advance to the point whereby the viability of the fetus is pushed back to, say, 20 weeks.^96^ Indeed, we can imagine a hypothetical case where the AAPTs have become so advanced that the point of viability is pushed back to 12 weeks or, perhaps, even earlier. What is doing the intuitive moral and legal work in these cases is (a) the health and wellbeing of the pregnant woman experiencing an unwanted pregnancy, and (b) the point of viability of the fetus. But once these features have been sufficiently transformed, such that advancements in AAPTs make ectogestation a safe and reasonable alternative to lethal abortions, one may draw the inference that a corresponding legal change is legally required. But what reason is there to think that any courts would converge on anything like a consensus concerning the legal meaning of viability or how this alleged legal threshold would be applied from one jurisdiction to another? While some might maintain that viability just means the fetus can be supported independent of the human womb, others have argued that if the notion of ‘viability’ holds any legal water at all, then it is best understood as meaning that the fetus is in some important respect capable of adapting to the external environment.^97^ So, given the ambiguity and lack of consensus regarding what viability is, it simply is not clear whether any court would actually use the notion of fetal viability, let alone consistently apply the concept in case law—at best this remains an open question. Thus, the objection can be avoided.

*Fourth Objection*: It might be claimed that appealing to systemic misogyny is just another way of saying that prohibitions on lethal abortions would violate a pregnant person’s right to autonomy. So, there is something inconsistent involved in the argument I have defended.

*Response*: Systemic misogyny might involve a violation of a pregnant person’s right to autonomy, if autonomy is broadly construed to include the role one’s cognitive abilities play in determining their own life narrative, insofar as misogyny is a form of thought police or gaslighting that attempts to control what the authoritative and meaningful narrative of one’s life is.^98^ But this is not the same as a violation of one’s more narrowly defined right to bodily autonomy. This narrow approach to bodily autonomy is primarily concerned with one’s decisions about what to do with and to their own body, which is naturally construed in terms of one’s medical choices. But the autonomy involved in systemic misogyny is not restricted to one’s medical choices about their body. As Dworkin (1993) says regarding this alternative view of autonomy, it ‘encourages and protects people’s general capacity to lead their own lives out of a distinctive sense of their own character, a sense of what is important to and for them’.^99^ While this broader approach includes medical decisions, it also outstrips them. Here is what Dworkin says regarding autonomy:

Recognizing an individual right to autonomy makes self-creation possible. It allows each of us to be responsible for shaping our own lives according to our own coherent or incoherent—but, in any case, distinctive—personality. It allows us to lead our own lives rather than be led along by them, so that each of us can be, to the extent a scheme of rights can make this possible, what we have made ourselves.^100^

And if this alternative view of autonomy is plausible and since it outstrips the more commonly accepted and narrowly defined view of one’s right to bodily autonomy, the above objection is guilty of equivocating between these different senses of ‘autonomy’. Thus, the objection can be avoided.

But the *Real* problem with this objection is that it mistakenly assumes that the issue of abortion involves a problem of conflicting rights between a pregnant person’s right to bodily autonomy versus a fetus’s alleged right to life.^101^ While this approach to the alleged problem of abortion may be widely accepted by some, it is not the only approach available.^102^ In reality, a more accurate approach does not view the issue of abortion as something (a problem) that needs to be solved.^103^ Rather, it treats the issue of abortion as an internal and private matter that people experiencing an unwanted pregnancy face—that is, an internal assessment of psychological and normative reasons. Moreover, treating abortion as a problem of conflicting rights is empirically inadequate.^104^ If the orthodox approach to abortion (i.e., the rights-based approach) is true, then we should expect that women would report that the possibility of using AAPTs in the form of ectogestation would solve this alleged problem. But according to qualitative research on the issue, the exact opposite has been found.^105^ For example, one influential study involving some self-identified Pro-Life and Pro-Choice individuals, some of whom had an abortion, both groups rejected the claim that ectogestation would be a reasonable solution to the problem of unwanted pregnancy. And in both cases, the reason was at least partly grounded in the idea that a pregnant person was thought to have certain inescapable responsibilities to the fetus. For instance, those who opposed abortion reported that the problem is not only grounded in whether a fetus has a right to life, it is also generated by the view that a pregnant person ought to gestate *and* raise the fetus.^106^

The reports of those who support abortion suggest that if a pregnant person cannot accept all of their parental responsibilities, then the best course of action is to abort the fetus.^107^ Here is how Cannold summarizes these findings:

Thus, while for women in favor of abortion rights, ectogenesis is problematic because it preserves the life of the fetus, and with that life, a woman’s maternal responsibilities, for women opposed to abortion rights, ectogenesis is a concern because it continues to enable women to avoid their responsibility to gestate, bear and raise the children they conceive.^108^

Studies like this provide *prima facie*, defeasible evidence in support of the view that individuals on both sides of the debate reject ectogestation as a legitimate solution to the alleged ‘problem’ of abortion.^109^ So, in short, even if there is no ‘moral’ right to the death of the fetus, this does not mean that societies ought to implement new legal prohibitions on a pregnant person’s ability to safely obtain an abortion that results in the death of the fetus.

## V. CONCLUDING REMARKS

We have seen that many philosophers have been interested in various moral and legal implications that AAPTs can conceivably introduce once ectogestation provides a safe and widely available alternative to lethal forms of abortion. And given that *Dobbs v. Jackson* overturned the precedent set by *Roe v. Wade* and *Planned Parenthood v. Casey* in the United States, questions regarding ectogestation will arguably continue to be of central philosophical importance and interests for issues concerning the legal status of abortion. The argument defended in this article represents a philosophically significant reason for why AAPTs can intersect with or even determine abortion policy. And it was shown that societies should not implement legal prohibitions on lethal abortions because such laws are systemically misogynistic and harmful. Given the uncertainty interjected into the legal landscape by *Dobbs*, and the need for philosophers, legal scholars, and medical practitioners to reflect on emerging medical technologies like AAPTs, future work concerning ectogestation will continue to be of importance for the ethics of reproduction.

## Footnotes

1
*Dobbs v. Jackson Women’s Health Organization*, 597, U.S. (2022).

2
*Roe v. Wade*, 113, U.S., (1973); *Casey v. Planned Parenthood*, 112, U.S. (1992).

3 Sarah Langford, *An end to abortion? A feminist critique of the ‘ectogenetic solution’ to abortion*, 31(4) Women’s Studies International Forum, 263 (2008); Frida Simonstein & Michal Mashiach-Eizenberg, *The Artificial Womb: A Pilot Study Considering People’s Views on the Artificial Womb and Ectogenesis in Israel*, 18(1) Cambridge Quarterly of Healthcare Ethics, 87 (2009); Tuija Takala, *Human Before Sex? Ectogenesis as a Way to Equality*, in Reprogen-ethics and the future of gender. International Library of Ethics, Law, and the New Medicine, vol 43. 187 (Frida Simonstein ed., 2009); Christine Overall, *Rethinking Abortion, Ectogenesis, and Fetal Death*, 46(1) Journal of Social Philosophy, 126 (2015); Michael Hawking, *The Viable Violinist*, 30(5) Bioethics, 312, (2015); Eric Mathison & Jeremy Davis, *Is There a Right to the Death of the Foetus?*, 31(4) Bioethics, 313, (2017); Joona Räsänen, *Ectogenesis, abortion and a right to the death of the fetus*, 31(9) Bioethics, 697 (2017); Perry Hendricks, *There is no right to the death of the fetus*, 32(6), Bioethics, 395, (2018); Christopher Kaczor, *Ectogenesis and a right the death of the prenatal human being: A reply to Räsänen*, 32(9) Bioethics, 634, (2018); Bruce Blackshaw & Daniel Rodger, *Ectogenesis and the case against the right to the death of the foetus*, 33(1) Bioethics, 76, (2019); Kathryn Mackay, *The ‘tyranny of reproduction’: Could ectogenesis further women’s liberation?*, 34(4) Bioethics, 346, (2020); Elizabeth Romanis, *Is ‘viability’ viable? Abortion, conceptual confusion and the law in England and Wales and the United States*, 7(1) Journal of Law and the Biosciences, 1, (2020); Lydia Di Stetano et al., *Ectogestation ethics: The implications of artificially extending gestation for viability, newborn resuscitation and abortion*, 34(4) Bioethics, 371, (2020); Johanna Eichinger & Tobias Eichinger, *Procreation machines: Ectogenesis as reproductive enhancement, proper medicine or a step towards posthumanism?*, 34(4) Bioethics, 385, (2020); Laura Kimberly et al., *Equitable access to ectogenesis for sexual and gender minorities*, 34(4) Bioethics, 338, (2020); Elselijn Kingma & Suki Finn, *Neonatal incubator or artificial womb? Distinguishing ectogestation and ectogenesis using the metaphysics of pregnancy*, 34(4) Bioethics, 354, (2020); Claire Horn, *Abortion Rights after Artificial Wombs: Why Decriminalisation is Needed Ahead of Ectogenesis*, 29(1) Medical Law Review, 80, (2021); Christopher Stratman, *Ectogestation and the Problem of Abortion*, 34Philosophy & Technology, 683–700, (2020).

4 Alejandro Aguilera-Castrejon et al., *Ex utero mouse embryogenesis from pre-gastrulation to late organogenesis*, 593(7857) Nature, 119, (2021); Gretchen Vogel, *No uterus, no problem: Mouse embryos grown in bottles from organ and limbs*. Science, (2021); Felix De Bie et al., *Ethics Considerations Regarding Artificial Womb Technology for the Fetonate*, 1 The American Journal of Bioethics, (2022); Antonio Regalado, *A mouse embryo has been grown in an artificial womb—humans could be next*, MIT Technology Review, https://www.technologyreview.com/2021/03/17/1020969/mouse-embryo-grown-in-a-jar-humans-next/ (date accessed March 24, 2023).

5
Peter Singer & Deane Wells, The Reproduction Revolution: New Ways of Making Babies, (1984); Christine Overall, Ethics and Human Reproduction (1987); Mathison & Davis, *supra* note 3; Blackshaw & Rodger, *supra* note 3; Hendricks, *supra* note 3; Kaczor, *supra* note 3; Stratman, *supra* note 3.

6 Mathison & Davis, *supra* note 3; Blackshaw & Rodger, *supra* note 3; Hendricks, *supra* note 3; Kaczor, *supra* note 3; Stratman, *supra* note 3.

7 Leslie Cannold, *Women, Ectogenesis and Ethical Theory*, 12(1), Journal of Applied Philosophy, 55, (1995); Partridge et al., *An extra-uterine system to physiologically support the extreme premature lamb*, 8(1) Nature Communications, 15,112, (2017); Overall, *supra* note 3, (2015); Räsänen, *supra* note 3; Phillip Wozniak & Ashley Fernandes, *Conventional revolution: the ethical implications of the natural progress of neonatal intensive care to artificial wombs*, Journal of Medical Ethics, (2020), https://jme.bmj.com/content/47/12/e54; Stav Dimitropoulos, *Artificial Wombs Are Getting Closer to Reality for Premature Babies*, leaps.org, https://leaps.org/artificial-wombs-are-getting-closer-to-reality-for-premature-babies/ (accessed August 10, 2022); Regalado, *supra* note 4.

8 Amel Alghrani, *The Legal and Ethical Ramifications of Ectogenesis*, 2(1) Asian Journal of WTO and International Health Law and Policy, 189, (2007); Emily Jackson, *Degendering Reproduction?*, 16(3) Medical Law Review, 246, (2008); Elizabeth Romanis, *Artificial Womb Technology and the Choice to Gestate Ex Utero: Is Partial Ectogenesis the Business of the Criminal Law?*, 28(2) Medical Law Review, 342, (2019); Horn *supra* note 3.

9 While the primary focus in this article is on various jurisdictions in western societies, the argument to be developed in what follows can be extended to non-western societies, insofar abortion bans are generally misogynistic and harmful.

10 For a recent overview of the complications and ethical implications involved in the development of artificial womb technologies, see eg, De Bie et al., *supra* note 4.

11 Elizabeth Romanis, *Artificial Womb technology and the frontiers of human reproduction: Conceptual differences and potential implications*, 44(11) Journal of Medical Ethics, 753, (2018).

12 Elizabeth Romanis, *Artificial womb technology and clinical translation: Innovative treatment or medical research?*, 34(4) Bioethics, 392–402, (2019); see also, Romanis, *supra* note 3.

13 Nicholas Colgrove, *Subjects of ectogenesis: Are ‘gestatelings’ fetuses, newborns or neither?*, 45(11), Journal of Medical Ethics, 723–726, (2019). And for further discussion of this distinction and the relevant moral implications see also, Daniel Rodger, *Why Ectogestation Is Unlikely to Transform the Abortion Debate: a Discussion of ‘Ectogestation and the Problem of Abortion’*, 34 Philosophy & Technology, 1929–1935, (2021).

14 In general, I agree with Romanis’ motivation for using the term ‘gestateling’ to distinguish between the entity gestating in a human womb and an entity gestating independent of the human womb. But the primary concern of this article is not to advance this peripheral debate. So, I will not adopt this terminology in what follows. Instead I will remain neutral.

15 Some prefer to use the term ‘ectogenesis’ while others prefer the term ‘ectogestation’. But it is important to note that these terms are not describing a significantly different biological process. Both terms refer to all forms of gestation independent of a human womb. The primary difference in terminology is the result of various metaphysical issues and debates that have emerged in the literature regarding AAPTs. For further discussion of these and related issues, see eg, Scott Gelfand and John Shook, Artificial Womb Technology and the Future of Human Reproduction (2006); see also, Alghrani, *supra* note 6; Kingma & Finn, *supra* note 3; and Di Stefano et al., *supra* note 3.

16 Romanis, *supra* note 3, at 11–12.

17 Romanis, *supra* note 3, at 13.

18 Unless otherwise specified, I will treat the phrase ‘abortion resulting in fetal death’ as interchangeable with ‘lethal abortion’. And occasionally, I will simply refer to ‘lethal abortion’ and ‘non-lethal abortion’. I do not mean any malice or to imply any significant different with this terminological choice. But someone might complain by suggesting that most of the time when the term ‘abortion’ is used it refers to abortions that in fact do result in fetal death. So, one might reasonably think that our commonsense usage of the term ‘abortion’ just is the termination of one’s pregnancy by killing the fetus. While I accept this point about our common usage of the term, I doubt that this is simply a verbal dispute. This is because the term alone does not logically or conceptually entail that the fetus must die to terminate one’s pregnancy. For further discussion of this point, see eg, Mathison & Davis, *supra* note 3.

19 Romanis, *supra* note 3, at 13.

20 Someone will likely object to these conditions insofar it is very hard to see how AAPTs could be less burdensome than abortion is currently. For a discussion of why some feminist scholars have stressed the importance of recognizing the physical burdens of abortion when attempting to establish the permissibility of abortion, see eg, Elizabeth Romanis & Claire Horn, *Artificial Wombs and the Ectogenesis Conversation: A Misplaced Focus? Technology, Abortion, and Reproductive Freedom*, 13(2) International Journal of Feminist Approaches to Bioethics, 174–194 (2020).

21 Rodger, *supra* 13.

22 For further discussion of this line of argument, see eg, Evie Kendal, *Pregnant people, inseminators and tissues of human origin: How ectogenesis challenges the concept of abortion*, 38(2) Monash Bioethics Review, 197–204 (2020); Alghrani, *supra* note 8; Jackson, *supra* note 8; Romanis & Horn, *supra* note 20; Rodger, *supra* note 13; Elizabeth Romanis et al., *Reviewing the Womb*, 47(12) Journal of Medical Ethics, 820–829 (2020); Elizabeth Romanis, *Abortion & ‘Artificial Wombs’: Would ‘Artificial Womb’ Technology Legally Empower Non-Gestating Genetic Progenitors to Participate in Decisions about How to Terminate Pregnancy in England and Wales?*, 8(1) Journal of Law and the Biosciences, (2021).

23 We need not focus only on inequities that occur in the United States, since many authors from all over the globe have raised similar worries regarding the potential moral and legal consequences of ectogestation.

24 Amel Alghrani, Regulating Assisted Reproductive Technologies: New Horizons (2007); Alghrani, *supra* note 8; Jackson, *supra* note 8; Romanis, *supra* note 3; Romanis, *supra* note 23; Horn, *supra* note 3.

25 I will not mention or discuss them at any length.

26 The reader should be aware that there are various important questions and disputes pertaining to ectogestation and fetal neonate life support that I have not addressed. For a discussion of these, see eg, De Bie et al., *supra* note 4. It is also worth mentioning that many of these questions and disputes focus on various feminist perspectives, which may partly determine or influence how one might approach the arguments discussed in what follows. See eg, Julien Murph, *Is Pregnancy Necessary? Feminist Concerns About Ectogenesis*, 4(3) Hypatia, 66–84 (1989); Soran Reader, *Abortion, Killing, and maternal Moral Authority*, 23(1) Hypatia, 132–149 (2008); Claire Horn, *Ectogenesis Is for Feminists*, 6(1) Catalyst: Feminism, Theory, Technoscience (2020); Jackson, *supra* note 8; Langford, *supra* note 3; Takala, *supra* note 3; (2009); Romanis, *supra* note 11; Colgrove, *supra* note 13; Kimberly, *supra* note 3; Eichinger & Eichinger, *supra* note 3; Mackay, *supra* note 3. However, the reader should note that, for the purpose of this article, I will focus on the legal status of lethal abortions only, assuming the advent of ectogestation makes it possible to terminate a pregnancy without killing the fetus in the process.

27 Judith Thomson, *A Defense of Abortion*, 1(1) Philosophy & Public Affairs, 47–66, (1971). According to Thomson, Minimally Decent Samaritan laws would compel individuals to meet a general standard of minimally decent treatment to others. But Good Samaritan Laws are stricter, insofar as they compel individuals to go out of their way and, perhaps, risk their own wellbeing in order to provide help to others. And it is worth mentioning here that Thomson’s insights in this regard are particularly reveling given the United States Supreme Court’s recent ruling that overturned of Roe v. Wade.

28
*Id*. at 64.

29
*Id*. at 63–64.

30 For a discussion of why someone might think that Thomson misrepresents the original and correct meaning of the Good Samaritan analogy, and why pro-lifers might consider themselves to be morally required to be Good Samaritans, see eg, Bruce Blackshaw, *Is pregnancy really a Good samaritan act?*, 27(2) Christian Bioethics: Non-Ecumenical Studies in Medical Morality, 158–168, (2021). The astute reader will note that the argument I defend here does not hinge on an alleged misrepresentation of the Good Samaritan analogy.

31 Hypothetical Syllogism from 1 & 2.

32 6 immediately follow from the truth of 4 & 5.

33 Modus Ponens 3 & 6.

34 Thomson, *supra* note 27; Norman Gillespie, *Abortion and Human Rights*, 87(3) Ethics, 237–243, (1977); Marry Ann Warren, *On the moral and Legal Status of Abortion*, in The problem of Abortion, Feinberg ed., (1984); Singer & Wells, *supra* note 5; Overall, *supra* note 5.

35 But for examples of those who might deny this claim, see eg, Alghrani, *supra* note 8; Jackson, *supra* note 8; Romanis, *supra* note 22, note 24; Horn, *supra* note 3.

36 Cannold, *supra* note 7, at 62; emphasis added.

37
*Id*. at 62; emphasis in the original.

38 For discussion of similar points regarding the requirement of being a ‘good mother’, see eg, Romanis, *supra* note 22.

39 It is important to recognize that the issue here involves both legal and moral dimensions.

40 Kate Manne, Down Girl, 32, (2018).

41
*Id.* at 33–34; emphasis in the original.

42
*Id*. at 33; emphasis in the original.

43 Indeed, as early as 1974, well before Manne’s ameliorative analysis of systemic misogyny, various feminist authors were articulating an approach to misogyny that could be construed in systemic terms along the same lines that Manne offers. See eg, Andrea Dworkin, Woman Hating (1974); Marilyn Frye, The politics of reality: Essays in feminist theory (1983); Catharine MacKinnon, Only Words (1993).

44 Lynne Tirrell, *Toxic Misogyny and the Limits of Counterspeech*, 87(6) Fordham Law Review, 2433–2452, (2019).

45 The Scottish Government, *Misogyny-a human rights issue*, https://www.gov.scot/publications/misogyny-human-rights-issue/ (accessed November 19, 2022); see also, The Scottish Government, Misogyny a human rights issue, Girls’ Attitudes Survey, https://www.gov.scot/publications/misogyny-human-rights-issue/pages/5/ (accessed November 19, 2022).

46
*Id*. See eg, section 4.1 of the full report: https://www.gov.scot/publications/misogyny-human-rights-issue/.

47 I have focused primarily on misogyny as a property of western societies, but it is worth mentioning that these arguments can be generalized to apply to non-western societies too.

48 Tirrell, *supra* note 44, at 2537.

49 David Gritten, *Iran hands out more death sentences to anti-government protesters—BBC,*  https://www.bbc.com/news/world-middle-east-63648629 (accessed November 19, 2022); Deepa Parent and Ghoncheh Habibiazad*, Iranian forces shooting at faces and genitals of female protesters, medics say—Guardian News Media*, https://www.theguardian.com/global-development/2022/dec/08/iranian-forces-shooting-at-faces-and-genitals-of-female-protesters-medics-say (accessed March 13, 2023).

50 Tara Sepehri Far, *Woman Dies in Custody of Iran’s ‘Morality Police’ Unexplained Death Underscores Need to Address Violence Against Iranian Women—Human Rights Watch*, https://www.hrw.org/news/2022/09/16/woman-dies-custody-irans-morality-police (accessed November 19, 2022).

51 Of course, there are many countries in the Middle East that have far more liberal laws than those in Iran. I do not want to suggest that every government in the Middle East is guilty of such cruelty. Rather, my point is simply to draw attention to the harms that result from patriarchal social institutions and the misogynistic laws operative in Iran. For example, there have been numerous reports of deliberate poisoning that killed hundreds of schoolgirls: See e.g., Iran: Deliberate poisoning of schoolgirls further evidence of continuous violence against women and girls—United Nations Human Rights Report: https://www.ohchr.org/en/press-releases/2023/03/iran-deliberate-poisoning-schoolgirls-further-evidence-continuous-violence (accessed March 13, 2023).

52 Iran: Abusive forced veiling laws police women’s lives—Amnesty International article, https://www.amnesty.org/en/latest/campaigns/2019/05/iran-abusive-forced-veiling-laws-police-womens-lives/ (accessed October 11, 2022).

53 Manne, *supra* note 40, at 33.

54 Karen Knudsen, *Impact of the Dobbs v Jackson Women’s Health Organization Ruling on Patients With Cancer—The ASCO Post*, https://ascopost.com/issues/august-25-2022/impact-of-the-dobbs-v-jackson-women-s-health-organization-ruling-on-patients-with-cancer/ (accessed November 19, 2022).

55 Michele Goodwin (witness testimony), *‘The Impact of the Supreme Court’s Dobbs Decision on Abortion Rights and Access Across the United States’—*Congress.gov, https://www.congress.gov/event/117th-congress/house-event/114986?s=1&r=16 (accessed March 13, 2023); Michele Goodwin, ‘I Was Raped by My Father. An Abortion Saved My Life.’—The New York Times, https://www.nytimes.com/2021/11/30/opinion/abortion-texas-mississippi-rape.html (accessed March 13, 2023); Samantha Artiga, *What are the Implications of the Overturning of Roe v. Wade for Racial Disparities?—Kaiser Family Foundation*, https://www.kff.org/racial-equity-and-health-policy/issue-brief/what-are-the-implications-of-the-overturning-of-roe-v-wade-for-racial-disparities/ (accessed March 13, 2023); Keon Gilbert et al., *Dobbs, another frontline for health equity—Brookings*, https://www.brookings.edu/blog/how-we-rise/2022/06/30/dobbs-another-frontline-for-health-equity/ (accessed March 13, 2023); Cecilia Lenzen, *Facing higher teen pregnancy and maternal mortality rates, Black women will largely bear the brunt of abortion limits—The Texas Tribune*, https://www.texastribune.org/2022/06/30/texas-abortion-black-women/#:∼:text=Facing%20higher%20teen%20pregnancy%20and,their%20health%20and%20financial%20risks (accessed March 13, 2023); Katy Kozhimannil et al., *Abortion Access as a Racial Justice Issue*, 387(17) New England Journal of Medicine, 1537–1539, (2022); Räsänen et al., *Does Overruling Roe Discriminate against Women (of Colour)?*, 48(12) Journal of Medical Ethics, 952–956, (2022); Elizabeth Romanis. *The End of (Reproductive) Liberty as We Know It: A Note on Dobbs v. Jackson Women’s Health 597 USC__,* Medical Law International, https://journals.sagepub.com/doi/10.1177/09685332231154562 (accessed March 31, 2023).

56 Knudsen, *supra* note 55.

57 I want to thank an anonymous reviewer for the importance of taking this point of consideration seriously.

58 Jessica Winter, *The Dobbs Decision Has Unleashed Legal Chaos for Doctors and Patients—The New Yorker*, https://www.newyorker.com/news/news-desk/the-dobbs-decision-has-unleashed-legal-chaos-for-doctors-and-patients#:∼:text=News%20Desk-,The%20Dobbs%20Decision%20Has%20Unleashed%20Legal%20Chaos%20for%20Doctors%20and,ambiguous%20language%20and%20glaring%20omissions (accessed November 19, 2022).

59 For a discussion of how Good Samaritan laws could potentially impact the medical decisions that physicians routinely face, see e.g., Nicolas Northcut, *Is the Good Samaritan really good? A look into the possible harm caused by current Good Samaritan laws*, 9(1) Journal of Biosecurity, Biosafety, and Biodefense Law, (2018). It is important to recognize that I make mention of such potential harms only to draw attention to the fact that biological females would not be the only people potentially harmed by the *Dobbs* decision. This is not meant to draw attention away from the systemic misogyny involved at the core of abortion bans.

60 David Cohen, et al., *Rethinking Strategy After Dobbs*, 75 Stanford Law Review Online, https://www.stanfordlawreview.org/online/rethinking-strategy-after-dobbs/ (accessed March 13, 2023); Goodwin *supra* note 53; Gilbert et al. *supra*, note 55; Kozhimannil et al., *supra* note 55; (2022); Artiga et al., *supra* note 55; Lenzen, *supra* note 55; Räsänen et al., *supra* note 55; Romanis *supra*, note 55.

61 Timothy Bella, *Woman says she carried dead fetus for 2 weeks after Texas abortion ban*—The Washington Post, https://www.washingtonpost.com/politics/2022/07/20/abortion-miscarriage-texas-fetus-stell/ (accessed November 19, 2022).

62 See eg, Edward Helmore, *10-year-old rape victim forced to travel from Ohio to Indiana for abortion—Guardian News and Media*, https://www.theguardian.com/us-news/2022/jul/03/ohio-indiana-abortion-rape-victim (accessed November 19, 2022).

63 For a discussion of related arguments against abortion bans grounded in systemic misogyny, see e.g., Romanis, *supra* note 22; Romanis et al., *supra* note 22.

64 Mathison & Davis, *supra* note 3; Räsänen, *supra* note 3.

65 Mathison & Davis, *supra* note 3; Hendricks, *supra* note 3; Kaczor, *supra* note 3; Blackshaw & Rodger, *supra* note 3; Stratman, *supra* note 3.

66 Räsänen, *supra* note 3.

67 Carl Wellman, *Rights: Systematic Analysis*, in Encyclopedia of Bioethics 2306 W. Reich (1995).

68 Thomson, *supra* note 28, at 66.

69 Someone may object by arguing that this moves far too fast since what is needed is more a realistic or likely situation where the AAPTs are available and there is still a significant risk to the fetus. Indeed, it could be reasonably argued that a much more likely situation is one where this occurs, rather than a situation where there is little or no risk to the fetus. While I agree that there are significant questions and problems to consider regarding how we get to a point where ectogestation is not a highly risky process for the fetus, in what follows these questions are not my primary concern. Indeed, proponents of this argument typically treat such scenarios as a thought experiment and I will do the same. Thus, since those who argue that ectogestation might end the abortion debate typically focus on cases where the process is not more invasive to the mother or risky to the fetus, these will be the sorts of cases I shall focus on. The morally significant question regarding the first premise is whether a woman’s right to bodily autonomy is violated.

70 Kaczor, *supra* note 3, at 634. It is worth mentioning that the term ‘prenatal human being’ is morally loaded. A reasonably substitute that is not morally questionable is ‘prenatal biological human fetus’ or just ‘fetus’.

71 Mathison & Davis, supra note 3, at 313–314.

72 Blackshaw & Rodger, *supra* note 3, at 76–77.

73 Warren, *supra* note 35, at 117.

74 Alghrani, *supra* note 8, note 22; Jackson, *supra* note 8; Romanis, *supra* note 3, note 22; Horn, *supra* note 3.

75 Blackshaw & Rodger, *supra* note 3, at 77.

76 But for reasons why one might be skeptical, see e.g., Roder, *supra* note 13.

77 For a discussion of why someone might think that a legitimate government would have an interest in preserving the life of a fetus, see e.g., Ronald Dworkin, Life’s Dominion: An Argument About Abortion, Euthanasia, and Individual Freedom (1993), pp. 168–170.

78 For discussion of the argument, see e.g., Romanis, *supra* note 3, at 10–16.

79 Romanis, *supra* note 3, at 6.

80
*Casey v. Planned Parenthood*, *supra* note 2; see also, Romanis, *supra* note 3, at 7–8.

81 Romanis, *supra* note 3, at 8.

82
*Id*. at 8.

83
*Id*. at 9–10.

84
*Id*. at 10.

85 These laws are sometimes called ‘trigger’ laws since they are designed to be triggered the moment the Supreme Court overturned *Roe v. Wade*.

86 Romanis, *supra* note 3, at 10.

87 It is also worth noting that these so-called heartbeat laws are morally problematic for various reasons. For instance, such laws undermine both medical expertise and a pregnant person’s ability to make informed medical decisions since in many cases the screenings required to detect fetal abnormalities do not occur prior to the sixth week of pregnancy. Indeed, in many cases the pregnant person is not even aware they are pregnant before the sixth week. And such laws establish a vigilante system of enforcement, which encourages private citizens to invade one’s privacy. See e.g., Michelle Haining et al., *The Unethical Texas heartbeat Law*, 42(5) Prenatal Diagnosis, 535–541, (2020). But for a discussion of why it may be a mistake to dismiss heartbeat laws without careful reflection and argument, see eg, Bruce Blackshaw, *Are heartbeat bills ethically defensible?*, Bioethics, 1–2, (2020).

88 Abortion Act (1967) § 1.

89 Abortion Act (1967) § 1 (1) (a)–(d).

90 Rodger, *supra* note 13, at 2.

91 For the sake of these examples, let us suppose that the pregnant woman relinquishes all parental responsibilities to the adoptive parents with the intent that they will care for and love the fetus as it is gestated until full term and beyond.

92 For further discussion of similar arguments, see eg, Anna Nelson, *Should Delivery by Partial Ectogenesis Be Available on Request of the Pregnant Person?*, 15(1) International Journal of Feminist Approaches to Bioethics, 1–26, (2022); Kendal, *supra* note 22; Romanis, *supra* note 3.

93 Again, at 24 weeks, once the fetus has been detached from the human womb and gestated in the artificial womb, the intention is that the Smiths will take on legal guardianship including all parental responsibilities and will provide a caring and loving environment while the fetus develops into a child and then an adult person.

94 For discussion of partial ectogestation on request, see e.g., Nelson, *supra* note 92; Romanis, *supra* note 8.

95 For further discussion of similar arguments, see e.g., Kendal, *supra* note 22; Romanis, *supra* note 3.

96 We are ignoring whether the advancement of AAPTs is likely in this case and simply stipulating that it is possible.

97 Romanis, *supra* note 3: Horn, *supra* note 3.

98 For a discussion of this broader approach to autonomy, see e.g., Dworkin, *supra* note 77, at chapter 8.

99
*Id*. at 224.

100
*Id*. at 224.

101 Thomson, *supra* note 27; Gillespie, *supra* note 34; Warren, *supra* note 34; Singer & Wells, *supra* note 5; Overall, *supra* note 5.

102 For an example of someone who argues that ethicists should reject this theoretical framework to the problem of abortion in favor of a theory that takes seriously a woman views and needs, see e.g., Cannold, *supra* note 7, at 63.

103 For a discussion of this point, see e.g., Romanis & Horn, *supra* note 20; Horn, *supra* note 3.

104 For a discussion of similar arguments, see e.g., Romanis & Horn, *supra* note 20; Horn, *supra* note 3.

105 See e.g., Cannold, *supra* note 7; see also Simonstein & Mashiach-Eizenberg, *supra* note 3.

106 See e.g., Cannold, supra note 7 at 62.

107
*Id*. at 58.

108
*Id*. at 63.

109 The following modus tollens argument would be generated: (i) If a rights-based approach to the alleged problem of abortion is true, then we should expect that women would report that ectogestation would solve the alleged problem. But (ii) women on both sides of the debate reject the idea that ectogestation would solve the alleged problem. (iii) Therefore, the rights-based approach to the alleged problem of abortion is false.

